# Influence of Solution Treatment Time on Precipitation Behavior and Mechanical Properties of Mg-2.0Nd-2.0Sm-0.4Zn-0.4Zr Alloy

**DOI:** 10.3390/ma14175037

**Published:** 2021-09-03

**Authors:** Tao Ma, Sicong Zhao, Liping Wang, Zhiwei Wang, Erjun Guo, Yicheng Feng, Jingfang Li

**Affiliations:** 1 School of Material Science and Chemical Engineering, Harbin University of Science and Technology, Harbin 150000, China; mt9523mt@163.com (T.M.); lp_wang2003@126.com (L.W.); guoerjun@126.com (E.G.); fyc7806067@163.com (Y.F.); 2 Key Laboratory of Advanced Manufacturing and Intelligent Technology (MOE), Harbin University of Science and Technology, Harbin 150000, China; 3 Aero Engine Corporation of China, Harbin Dongan Engine Co., Ltd., Harbin 150000, China; xztvcn@163.com; 4 Key Laboratory of Functional Inorganic Material Chemistry (MOE), School of Chemistry and Materials Science, Heilongjiang University, Harbin 150000, China; fjlaaa.ok@163.com

**Keywords:** Mg-Nd-Sm-Zn-Zr alloy, solution treatment time, precipitate evolution, mechanical properties

## Abstract

The effect of solution treatment time on the microstructure and mechanical properties of aged the Mg-2.0Nd-2.0Sm-0.4Zn-0.4Zr (wt.%) alloy were investigated to give full play to the performance of the alloy. As the solution treatment time increased from 2 h to 12 h at 788 K, the grain size of the solution-treated alloy significantly increased, and the network-like *β*-Mg_12_(Nd, Sm, Zn) phase gradually dissolved into the *α*-Mg matrix. It should be noted that no obvious residual *β* phase can be observed when the solution treatment time was more than 8 h. After the solution-treated alloy was further aged at 473 K for 18 h, a large number of nanoscale precipitates were observed in the *α*-Mg matrix. The solution treatment time was 2 h, the *α*-Mg matrix mainly consisted of spherical-shaped and basal plate-shaped precipitates. Upon the increase of solution treatment time to 8 h, the key strengthening phases transformed from spherical-shaped precipitates and basal plate-shaped precipitates to prismatic plate-shaped *β*′ precipitates. The orientation relationship between *β*′ precipitates and *α*-Mg matrix was ($\bar{1}$10)*_β_*_′_ // (1$\bar{1}$00)*_α_* and [112]*_β_*_′_ // the [22$\bar{4}$3]*_α_*. Further increasing of solution treatment time from 8 h to 12 h, the key strengthening phases mainly were still *β*′ precipitates. The solution treatment of aged alloy was carried out at 788 K for 8 h, which achieved optimal ultimate tensile strength (UTS) of 261 ± 4.1 MPa, yield strength (YS) of 154 ± 1.5 MPa, and elongation of 5.8 ± 0.1%, respectively.

## 1. Introduction

Mg alloys are increasingly used in industrial applications, such as aerospace, electronic, and automobile applications according to their lightweight and remarkable mechanical properties [1,2,3]. It has been demonstrated that rare earth (RE) elements alloying is the most useful modification treatment to enhance mechanical properties of Mg alloys [4]. In recent years, numerous researches have been focused on Mg alloys with heavy RE elements, as an illustration of the Mg-Gd-Y-Zr [5,6,7], Mg-Gd-Y-Zn-Zr [8], Mg-Yb-Gd-Zn-Zr [9] and Mg-Gd-Y-Nd-Zn-Zr [10] alloys are developed, and these alloy usually exhibits excellent mechanical properties. However, the high content of heavy RE elements in matrix alloy increases the cost of alloys and reduces the advantages of lightweight structural alloy materials. To address these questions, rational use of light RE element could make it possible to develop a good combination of high strength and low-density Mg alloy. The maximum equilibrium solid solubility of Nd in *α*-Mg is approximately 3.62 wt.% at 825 K and down to nearly zero at 473 K [11]. The maximum equilibrium solid solubility of Sm in *α*-Mg is 5.8 wt.% and 0.4 wt.% at 815 K and 473 K, respectively [12]. Hence, the Mg-Nd-Sm series alloys [13] exhibit a significant age-hardening response, which greatly improves mechanical properties through heat treatment. Chang et al. [14] found that the Mg-3Nd-0.2Zn-0.4Zr alloy was achieved a peak of UTS by solution treated at 813 K for 6 h and subsequently aging treatment at 473 K for 10 h to 16 h. The value of UTS increased by about 130 MPa compared with the as-cast alloys. Additionally, Yuan et al. [15] reported that the precipitation strengthening of peak aged Mg-2.6Sm-1.3Gd-0.6Zn-0.5Zr alloy was the largest contributor to strength about 67%. The precipitation strengthening was mainly related to the morphology and number density of precipitates. As a pre-treatment before aging treatment, the solution treatment plays a critical role in aging precipitation behavior and mechanical properties. Its aim is to dissolve completely for the eutectic phases and obtain a supersaturated solid solution of alloying elements in *α*-Mg [16]. As the short of solution treatment time, the residual eutectic phases are distributed at the grain boundaries of solution-treated alloy, resulting in changes in the morphological distribution of the precipitates after aging treatment [17]. Or vice versa, the excessive solution treatment time leads to grain coarsening, and the mechanical properties of alloys are deteriorated seriously [18].

Current research mainly focuses on the improvement of performance via adjusting aging treatment parameters. A few reports about the influence of solution-treatment on the precipitation behavior and mechanical properties of aged Mg-RE alloys. Consequently, in order to give full play to the performance of the Mg-RE alloys and broaden its application fields, the influence of solution-treatment on the grain size, precipitates morphology, precipitates distribution, precipitates orientation relationship, mechanical properties, and tensile fractures of aged Mg-2.0Nd-2.0Sm-0.4Zn-0.4Zr alloys are studied in detail by adjusting the solution treatment time.

## 2. Materials and Methods

The as-cast Mg-2.0Nd-2.0Sm-0.4Zn-0.4Zr (wt.%) ingots were prepared from pure Mg ingots and pure Zn ingots (99.9 wt.%), Mg-30Nd (wt.%), Mg-25Sm (wt.%), and Mg-25Zr (wt.%) binary alloys. The experimental ingots were melted in steel crucible under a protective atmosphere of mixed SF_6_/CO_2_ with the ratio of 100:1 in an electrical resistance furnace. First, the pure Mg was melted at 993 K. When the temperature in the electrical resistance furnace raised to 1023 K, the Mg-30 wt.%Nd, Mg-25 wt.%Sm binary alloys and pure Zn were added into the melting. Whereafter, as the temperature of the alloy melt reached 1053 K, Mg-25 wt.%Zr binary alloy was added into the melting. Finally, the melting was poured into a steel mold preheated at 523 K. The solution treatment of as-cast alloy was performed at 788 K for different holding times (2 h, 4 h, 6 h, 8 h, 10 h, and 12 h), then the alloy was quenched in hot water at approximate 353 K to obtain the supersaturated solid solution. Subsequently, the solution-treated alloy was aged at 473 K for 18 h.

The optical microscope (OM, OLYMPUS-GX71, Olympus Co., Tokyo, Japan) was employed to characterize the microstructure of as-cast alloys and solution-treated alloys. For OM observation, the samples were mechanically ground, polished, and then etched using the picric acid solution [4]. The average grain size was determined through the linear intercept method from the low magnification OM images. The measuring results were based on the average of five images to ensure the accuracy of average grain size. The morphology and orientation relation of precipitates were observed by transmission electron microscope (TEM, JEM-2100, Japan Electron Optics Laboratory Co. Ltd., Tokyo, Japan). Specimens for TEM characterization were firstly mechanically ground to about 50 μm and then ion milling operating at 4 kV ion gun energy and 4° milling angle under a protective atmosphere of Ar. The tensile testing was conducted by using a material testing machine (MTS E44.304, MTS Systems Co., Eden Prairie, MN, USA) at room temperature under a strain rate of 1 × 10^−3^ s^−1^. The samples of tensile tests with a rectangular cross-section of 2 mm (±5 μm) × 5 mm (±5 μm) and a gauge length of 15 mm (±5 μm) were prepared by wire cutting machine. Tensile properties for the alloy were obtained based on the average of five tests to ensure the reproducibility of the experimental results. The fracture morphologies of aged samples were examined by scanning electron microscopy (SEM, Apreo C, Thermo Fisher Scientific Inc., Hillsboro, OR, USA).

## 3. Results and Discussion

### 3.1. Microstructure

**Figure 1:**
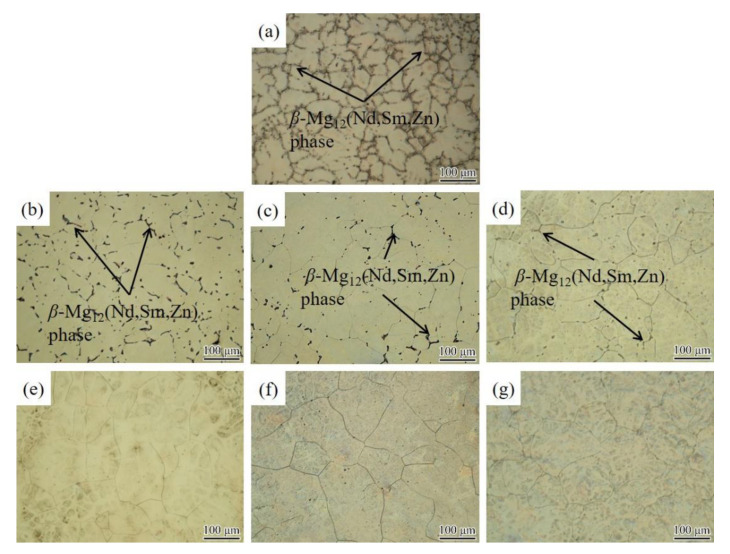
Optical images of Mg-2.0Nd-2.0Sm-0.4Zn-0.4Zr alloy. (**a**) as-cast alloy; the solution-treated alloys at 788 K for (**b**) 2 h, (**c**) 4 h, (**d**) 6 h, (**e**) 8 h, (**f**) 10 h and (**g**) 12 h.

**Figure 2:**
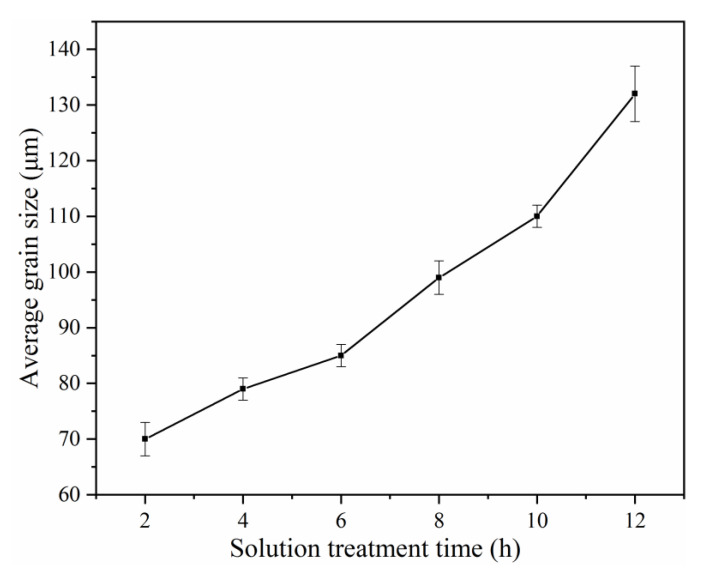
The average grain size of solution-treated alloy with different solution treatment time.

Figure 1 shows the OM images of the Mg-2.0Nd-2.0Sm-0.4Zn-0.4Zr alloy. The as-cast alloy consists of *α*-Mg matrix and network-like *β*-Mg_12_(Nd, Sm, Zn) phase along with grain boundaries [19], as shown in Figure 1a. The OM images of the solution-treated alloy at 788 K for 2 h to 12 h are shown in Figure 1b–g, respectively. The average grain size of solution-treated alloy with different solution treatment time is shown in Figure 2. Upon prolongation of solution treatment time, the alloy included three stages, under solution-treated stage (Figure 1b–d), peak solution-treated stage (Figure 1e), and over the solution-treated stage (Figure 1f,g). The microstructure of under solution-treated alloys contained an *α*-Mg matrix and a large number of undissolved *β* phases. The microstructure of peak solution-treated alloy and the over solution-treated alloys mainly consisted of an *α*-Mg matrix. However, the average grain size of over solution-treated stage alloys was higher than that of the peak solution-treated alloy. At the under solution-treated stage, the *β* phase dissolved into the *α*-Mg matrix deficiently. The solution treatment time was increased from 2 h to 6 h, and the average grain size achieved increment of 15 μm. The peak solution-treated alloy with an average grain size of 99 ± 3 μm occurred, and no obvious *β* phases were observed on the grain boundaries. It should be noted that the over solution-treated alloy grain size increased to 110 ± 2 μm and 132 ± 5 μm after solution treatment for 10 h and 12 h, respectively. It can be observed that the increment of average grain size of over solution-treated alloy was significantly higher than that of under solution-treated alloy. The main reason for grain rapid growth was the lack of *β* phase to hinder the grain boundary movement during elevated temperature solution treatment.

**Figure 3:**
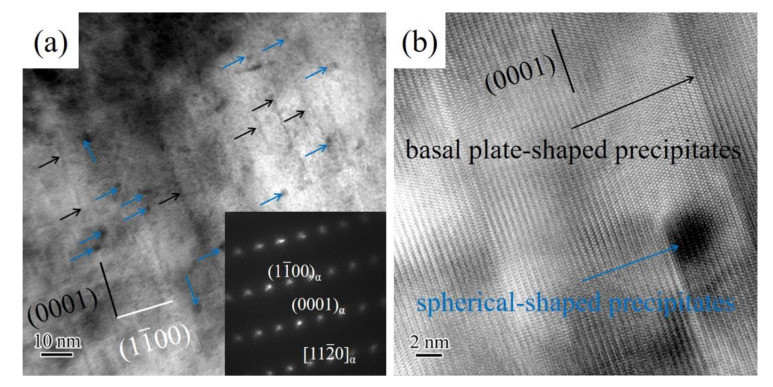
TEM images of aged Mg-2.0Nd-2.0Sm-0.4Zn-0.4Zr alloy with solution treatment for 2 h: (**a**) bright-field image and SAED patterns, (**b**) HRTEM of spherical-shaped precipitates and basal plate-shaped precipitates. The incident electron beam direction is parallel to the [11$\bar{2}$0]*_α_*.

In order to investigate the effect of solution-treated alloy microstructure on the precipitate evolution after the aging at 473 K for 18 h, TEM was used to observe and analyze morphology and distribution of precipitates of aged alloys with different solution treatment times (2 h, 8 h, and 12 h). The TEM bright-field images of the aged alloy with solution treatment for 2 h and corresponding selected area electron diffraction (SAED) patterns are shown in Figure 3a. It can be clearly seen that plate-shaped precipitates (indicated by the black arrow) and spherical-shaped (indicated by the blue arrow) occurred in an *α*-Mg matrix. The plate-shaped precipitates are parallel to the basal plane, and have a size of 10 nm to 20 nm in length along [1$\bar{1}$00]*_α_* direction. Figure 3b shows that the high-resolution transmission electron microscopy (HRTEM) of plate-shaped precipitates and spherical-shaped precipitates. The basal plate-shaped precipitates have a thickness of only about 1 nm. The morphology characters and orientation relationship of basal plate-shaped precipitates are similar to those basal precipitates in Mg-6Gd-Zn alloy [20] and Mg-10Gd-3Y-2Ag-0.4Zr alloy [21]. Usually, the addition of Zn (≥1 wt.%) was beneficial to the formation of basal precipitates [22,23]. The RE element of aged alloy with solution treatment for 2 h is mostly concentrated in the undissolved *β* phase, and the RE content in an *α*-Mg matrix is very low. The high radio of Zn/RE in the *α*-Mg matrix was favorited for the nucleation of basal plate-shaped precipitates. In addition, the spherical-shaped precipitates dispersed uniformly in an α-Mg matrix and had an approximate average diameter of 3 nm. The spherical-shaped precipitates clearly were observed in the present study are not reported in Mg-Nd-Sm series alloys. According to the research result of Zheng et al. [24], the formation of the spherical-shaped precipitates may attribute to the low concentration of RE element in the *α*-Mg matrix. The solute Nd and Sm atoms in α-Mg is insufficient for the subsequent transformation into *β*′ phase and *β*_1_ phase, which are conventional precipitate structures in Mg-Nd and Mg-Sm alloys [13,14]. Hence, the aged alloy with solution treatment for 2 h contains basal plate-shaped precipitates and spherical-shaped precipitates.

**Figure 4:**
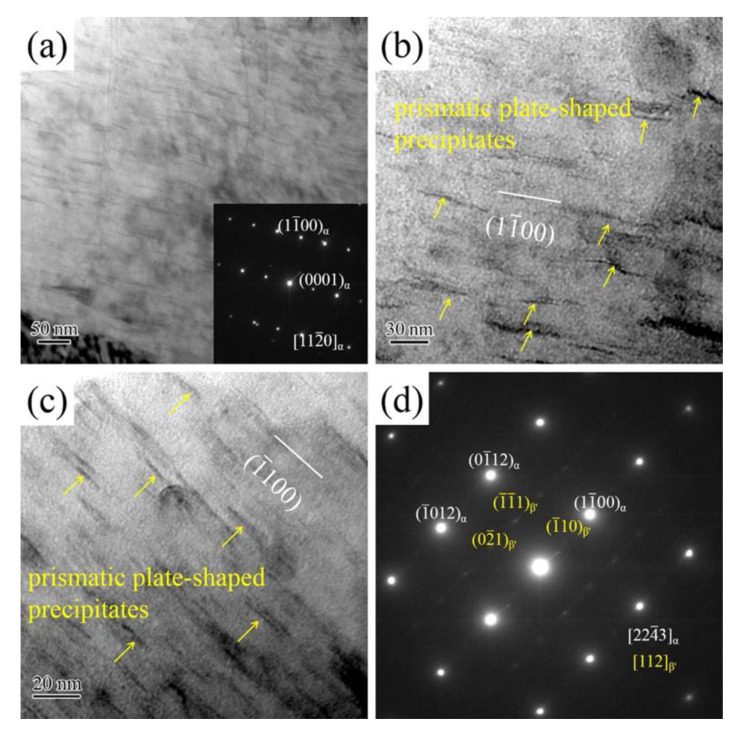
TEM bright-field images and corresponding SAED patterns of aged Mg-2.0Nd-2.0Sm-0.4Zn-0.4Zr alloy with solution treatment for 8 h: (**a**,**b**) the incident electron beam direction is parallel to the [11$\bar{2}$0]*_α_*; (**c**,**d**) the incident electron beam direction is parallel to the [22$\bar{4}$3]*_α_*.

Morphology of precipitates and corresponding SAED patterns of the aged alloy with solution treatment for 8 h, as shown in Figure 4. As the solution treatment time increased from 2 h to 8 h, the morphology and distribution of precipitates were changed obviously. The main precipitates transformed from basal plate-shaped precipitates and spherical-shaped precipitates to prismatic plate-shaped precipitates (indicated by the yellow arrow). According to Figure 4a,b, the prismatic plate-shaped precipitates have an average size of about 20 nm–50 nm in length along [1$\bar{1}$00]*_α_* direction, which distributed densely and uniformly in the *α*-Mg matrix under the incident electron beam direction parallel to [11$\bar{2}$0]*_α_* direction. Meanwhile, the prismatic plate-shaped precipitates parallel to the (1$\bar{1}$00)*_α_*. In order to further confirm the type of prismatic plate-shaped precipitates, the incident electron beam parallel to the [22$\bar{4}$3]*_α_* was carried out. The prismatic plate-shaped precipitates are still parallel to (1$\bar{1}$00)*_α_*. TEM field images and corresponding SAED patterns (Figure 4d) confirm that the prismatic plate-shaped precipitates are *β*′ precipitates. The orientation relationship between *β*′ precipitates and *α*-Mg matrix is ($\bar{1}$10)*_β_*_′_ // (1$\bar{1}$00)*_α_* and [112]*_β_*_′_ // [22$\bar{4}$3]*_α_*. With the increase of solution treatment time, the *β* phase was fully dissolved into the *α*-Mg matrix. The RE element content of supersaturated solid solution increased significantly. Hence, a large number of *β′* precipitates occurred in the *α*-Mg matrix.

**Figure 5:**
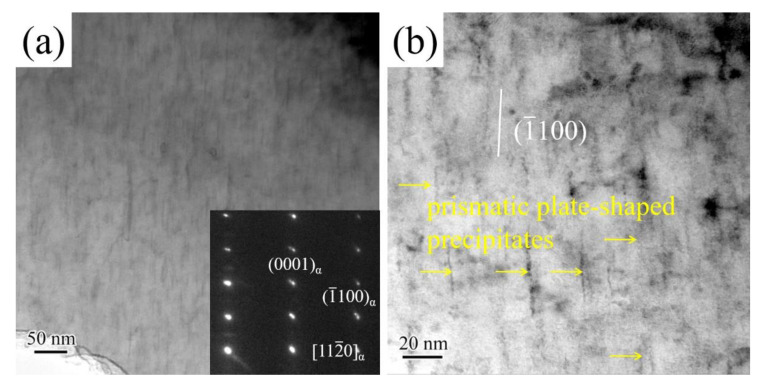
TEM bright-field image and SAED patterns of aged Mg-2.0Nd-2.0Sm-0.4Zn-0.4Zr alloy with solution treatment for 12 h, the incident electron beam direction is parallel to the [11$\bar{2}$0]*_α_*. (**a**) the low magnification TEM bright-field image, (**b**) the high magnification TEM bright-field image.

The TEM bright-field images of the aged alloy with solution treatment for 12 h and corresponding SAED patterns are shown in Figure 5. The main precipitates were prismatic plate-shaped precipitates (indicated by yellow arrow), which have an average of about 20 nm–50 nm in length along [1$\bar{1}$00]*_α_* direction. According to the morphology and orientation relationship of the precipitates, the prismatic plate-shaped precipitates were still *β*′ precipitates. Meanwhile, a small number of spherical-shaped precipitates were observed in the α-Mg matrix. The number of spherical-shaped precipitates was lower than that of aged alloy with solution treatment for 2 h. Although the solution treatment time further increased from 8 h to 12 h, the content of the RE element in the supersaturated solid solution keeps at the same level. Hence, compared to the peak solution-treated alloy, the precipitation behavior of the over solution-treated alloy was almost unchanged during the aging treatment.

### 3.2. Mechanical Properties

**Figure 6:**
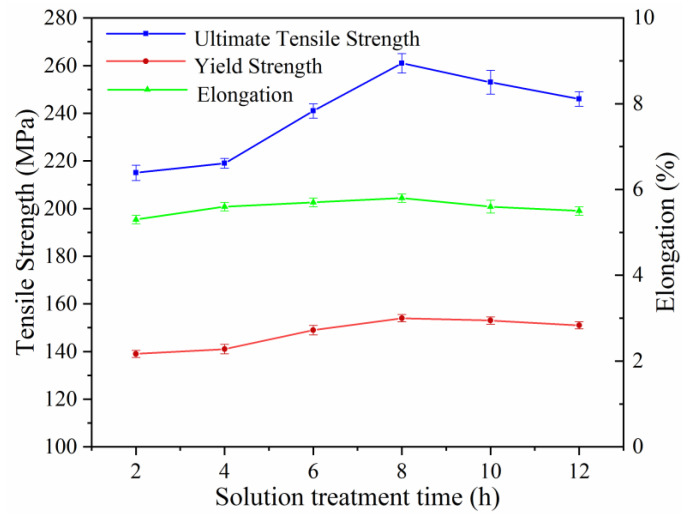
Mechanical properties of the aged Mg-2.0Nd-2.0Sm-0.4Zn-0.4Zr alloy with solution treatment for different times.

The relationship between the solution treatment time and mechanical properties of the aged Mg-2.0Nd-2.0Sm-0.4Zn-0.4Zr alloy is shown in Figure 6. As the solution treatment time increased from 2 h to 8 h, the value of UTS raised from 215 ± 3.2 MPa to 261 ± 4.1 MPa significantly, the value of YS raised from 139 ± 1.5 MPa to 154 ± 1.5MPa, but the elongation was in the same level. It’s worth noting that the UTS and YS decreased a little with the solution treatment time further prolonged from 8 h to 12 h, the UTS of the alloy was 246 ± 3.1 MPa, which decreased by 5.7%, and the YS of the alloy was 151 ± 1.5 MPa, which decreased by 1.9% compared with the alloy with optimal tensile strength alloy. It demonstrated that the further solution treatment time after 8 h cannot supply any increase in strength.

The strengthening of Mg alloy occurred through grain refinement, precipitation strengthening, solution strengthening, and so on [25]. Especially in Mg-RE alloy, the strengthening of nanoscale precipitates was the most important way to enhance the performance [26]. The basal slip was the dominant slip mechanism in Mg alloys at room temperature, since its critical resolved shear stress (CRSS) was substantially lower than those of non-basal slip systems such as prismatic and pyramidal slips [27]. Hence, the resistance effect of precipitates in aged alloy relative to basal slip was the key factor affecting the mechanical properties of the alloy. The precipitates in the aged alloy with solution treatment for 2 h mainly comprised of spherical-shaped precipitates and basal plate-shaped precipitates. According to research results of Nie [28] show that the contribution of the nanoscale spherical-shaped precipitates and basal plate-shaped precipitates on hindering the basal slip is weaker than that of the prismatic plate-shaped precipitates. Moreover, a large number of residual *β* phases are distributed along the grain boundary of the alloy, which shows the irregular shape and sharp edge leading to stress concentration under loading. Accordingly, the mechanical properties of the aged alloy with solution treatment for 2 h was much lower than alloys with the peak solution treatment condition. The optimal solution treatment time for the alloy is 8 h according to the microstructure and tensile properties. After the peak solution treatment and subsequent aging treatment, numerous *β*′ precipitates were achieved. For plate-shaped *β*′ precipitates formed on prismatic planes of *α*-Mg, the CRSS increment of basal slip was significantly larger than that of spherical particles and basal plate-shaped precipitates. The CRSS increases substantially with an increase in the average size of precipitates. On the other hand, the RE element content in the *α*-Mg matrix of peak solution-treated alloy is much higher than that of alloy with solution treatment for 2 h. The precipitate response was further promoted during the aging process. Therefore, prismatic plate-shaped *β*′ precipitates with a high number in aged alloys greatly improved the strength. Upon the solution treatment time was prolonged to 12 h, the type of precipitates in aged alloys still was dominated by *β*′ precipitates. Compared to the aged alloy with the peak solution-treatment condition, it had a slight loss of tensile strength value. After the peak solution treatment time, further prolongation for solution treatment time resulting in the grain rapid coarsening. According to the Hall-Petch relationship, the strength of the alloy decreased with the increase of average grain size [26].

### 3.3. Fractography

**Figure 7:**
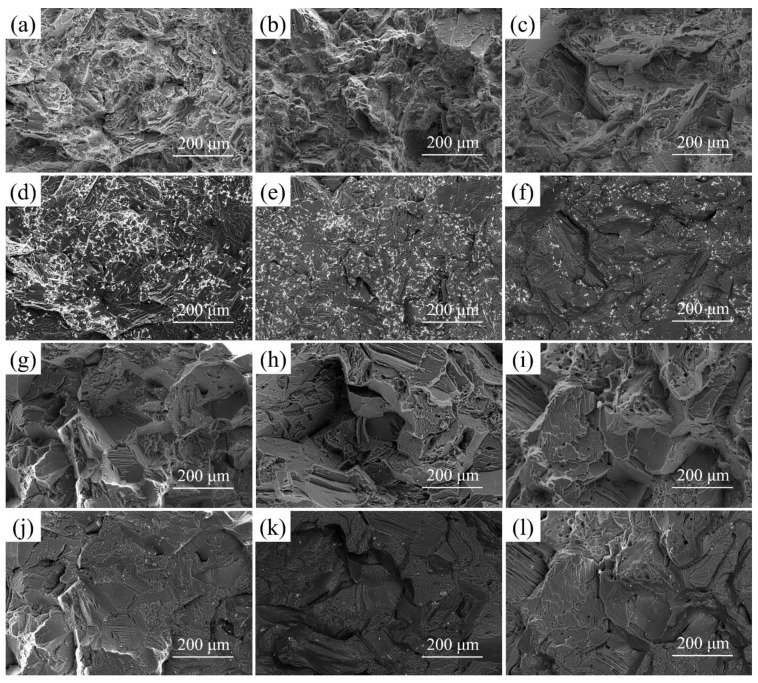
The tensile fracture microstructure of the aged Mg-2.0Nd-2.0Sm-0.4Zn-0.4Zr alloy by solution-treated at 788 K for (**a**,**d**) 2 h, (**b**,**e**) 4 h, (**c**,**f**) 6 h, (**g**,**j**) 8 h, (**h**,**k**)10 h, (**i**,**l**) 12 h; (**a**–**c**,**g**–**i**) are the secondary electron images, (**d**–**f**,**j**–**l**) are the back scattering electron images.

The typical tensile fracture microstructure of the aged Mg-2.0Nd-2.0Sm-0.4Zn-0.4Zr alloy under different solution treatment time, as shown in Figure 7. The fracture mode of the alloy is typical brittle cleavage fracture. The fracture microstructure of the alloy is mainly comprised of cleavage plane and tear ridge. It can be clearly seen that with the increase of solution treatment time, the size of the cleavage plane increases gradually. When the solution treatment time is less than 8 h, some residual *β* phases are observed on fracture microstructure, as shown in Figure 7d–f. With the solution treatment time exceed 8 h, the residual *β* phase disappears almost completely. The increase in the size of the cleavage plane is mainly attributed to the grain coarsening.

## 4. Conclusions

The influence of solution treatment time on microstructure and mechanical properties of Mg-2.0Nd-2.0Sm-0.4Zn-0.4Zr alloys was determined primarily:

1. The as-cast alloy mainly consists of *α*-Mg matrix and network-like *β*-Mg_12_(Nd, Sm, Zn) phase. With the increase of solution treatment time from 2 h to 12 h at 788 K, the grain size increases gradually, and the amount of *β* phase decrease gradually.
2. Precipitates morphologies in aged alloys include spherical-shaped precipitates, basal plate-shaped precipitates, and prismatic plate-shaped precipitates. The solution treatment time is carried out for 2 h, the *α*-Mg matrix mainly contains basal plate-shaped precipitates and spherical-shaped precipitates. As solution treatment time exceeds 8 h, a large number of prismatic plate-shaped *β*′ precipitates exist in the *α*-Mg matrix.
3. Upon solution treatment time increase from 2 h to 8 h, the improvement of mechanical properties is related to the key strengthening phase transforming from basal plate-shaped precipitates and spherical-shaped precipitates to prismatic plate-shaped *β*′ precipitates. Further increasing of solution treatment time to 12 h, the decrease of mechanical properties of aged alloys is attributed to grain coarsening.
4. The optimal solution treatment time for Mg-2.0Nd-2.0Sm-0.4Zn-0.4Zr alloy is 8 h, the YS and UTS of the alloy are 154 ± 1.5 MPa, and 261 ± 4.1 MPa, respectively, and the elongation rate is 5.8 ± 0.1%.

## Data Availability

The data presented in this study are available on request from the corresponding author.
